# Peripheral Arterial Disease in Women: an Overview of Risk Factor Profile, Clinical Features, and Outcomes

**DOI:** 10.1007/s11883-018-0742-x

**Published:** 2018-06-02

**Authors:** Qurat-ul-ain Jelani, Mikhail Petrov, Sara C. Martinez, Lene Holmvang, Khaled Al-Shaibi, Mirvat Alasnag

**Affiliations:** 1 Department of Cardiology, Bridgeport Hospital, Yale New Haven Health, New Haven, CT USA; 2 Department of Internal Medicine, Norwalk Hospital, Norwalk, CT USA; 3 Division of Cardiology, Providence St. Peter Hospital, Olympia, WA USA; 4 Department of Cardiology, Rigshospitalet, Copenhagen University Hospital, Copenhagen, Denmark; 5 Cardiac Center, King Fahd Armed Forces Hospital, Jeddah, Saudi Arabia

**Keywords:** Peripheral arterial disease, Women, Sex differences

## Abstract

**Purpose of Review:**

Peripheral arterial disease (PAD) is the third most common manifestation of cardiovascular disease (CVD), following coronary artery disease (CAD) and stroke. PAD remains underdiagnosed and under-treated in women.

**Recent Findings:**

Women with PAD experience more atypical symptoms and poorer overall health status. The prevalence of PAD in women increases with age, such that more women than men have PAD after the age of 40 years. There is under-representation of PAD patients in clinical trials in general and women in particular. In this article, we address the lack of women participants in PAD trials. We then present a comprehensive overview of the epidemiology/risk factor profile, clinical features, treatment, and outcomes.

**Summary:**

PAD is prevalent in women and its global burden is on the rise despite a decline in global age-standardized death rate from CVD. The importance of this issue has been underlined by the American Heart Association’s (AHA) “Call to Action” scientific statement on PAD in women. Large-scale campaigns are needed to increase awareness among physicians and the general public. Furthermore, effective treatment strategies must be implemented.

## Background

Peripheral arterial disease (PAD) remains a significant health concern across the globe. As of 2010, more than 200 million people worldwide are living with PAD, representing a 29% increased prevalence in low-middle income countries and 13% increase in high income countries [1, 2]. In the USA alone, PAD affects 8 million Americans aged > 40 years [3]. In the Reduction of Atherothrombosis for Continued Health (REACH) Registry, the cumulative end point of major cardiovascular events, vascular interventions, and hospitalization was significantly higher in patients with PAD than patients with coronary artery disease (CAD) [4]. PAD is associated with equal morbidity and mortality and economic costs as CAD and ischemic stroke [5, 6]. In a scientific statement on Women and PAD from the AHA in 2012 [7], Hirsch et al. noted the increased prevalence of PAD in adults ≥ 40 years of age, and highlighted the need for raising clinical awareness, focused treatment plans, and expanding research on PAD in women. Women have higher rates of asymptomatic/subclinical disease and the majority have atypical symptoms. They also have a poorer overall health status. Women with PAD suffer more from depression compared to women without PAD [8–15, 16•].

Overall, there is limited recruitment of patients with PAD in cardiovascular trials, especially women, minorities, and the elderly [17•]. For the purpose of this review, we will focus on the different aspects of PAD in women including data on representation in research studies, epidemiology, clinical features, and outcomes.

## Representation of Women in PAD Studies

**Fig. 1:**
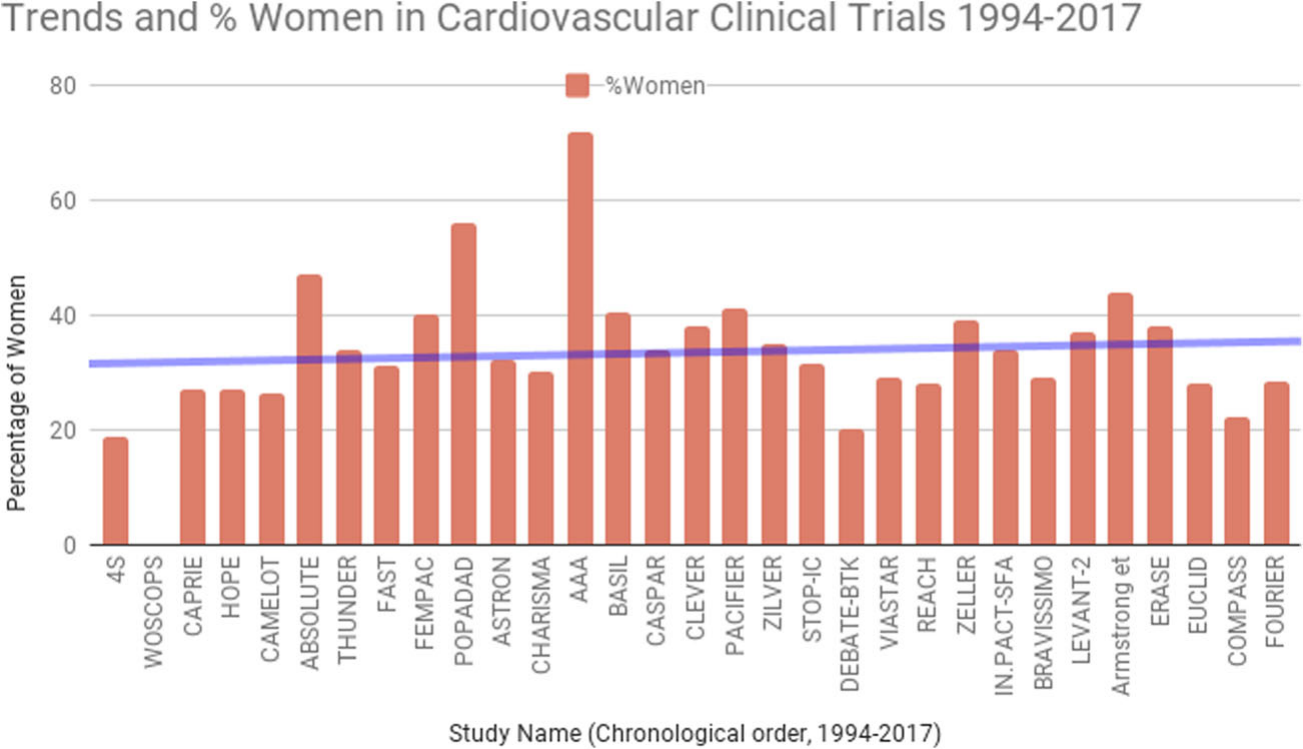
Trends and % women in cardiovascular clinical trials 1994–2017

Sex differences in PAD have been reported not only in prevalence, diagnosis, and clinical presentation but also in outcomes. Women continue to have variable enrollment in studies on PAD (Table 1 and Fig. 1). In more than half of these studies, women comprise < 35% of the whole study population. In the Nation Wide inpatient sample of patients with PAD, women comprised 41% of the study population. However, in randomized control trials (RCT) of vascular surgery, women represented only 22% [18]. In a systematic review of cardiovascular trials, which collectively enrolled 412,048 patients, only 27% of the total population were women [17•]. While enrollment of women has increased overall in clinical trials, it continues to lag behind their overall representation in this disease [19•].

**Table 1:** Brief overview of PAD trials and percentage of women enrolled

	Study design	Follow-up	Salient features	No. of patients	Men (%)	Women (%)	Outcomes
Pharmacotherapy							
CASPAR	RCT placebo	2 years	Patients undergoing vascular grafting as a treatment for PAD and 2 to 4 days after bypass surgery	851	66	34	Combination of clopidogrel plus ASA did not improve limb or systemic outcomes in the overall population of PAD patients requiring below-knee bypass grafting. Subgroup analysis: clopidogrel plus ASA conferred benefit in patients receiving prosthetic grafts
AAA	Intention-to-treat double-blind RCT	8.2 years	Patients free of clinical cardiovascular disease, recruited from a community health registry, with a positive ABI screening test	3350	28	72	Aspirin did not result in a significant reduction of vascular events among patients without clinical cardiovascular disease and a low ABI
POPADAD	RCT, double-blind, 2 × 2 factorial, placebo-controlled	6.7 years	Adults aged > 40 with type 1 or type 2 diabetes and an ABI of 0.99 or less but no symptomatic cardiovascular disease	1276	44	56	No benefit from either aspirin or antioxidant treatment on the composite hierarchical primary end points of cardiovascular events and cardiovascular mortality
CAPRIE	Double-blind RCT	1.91 years	Patients with atherosclerotic vascular disease	6452	73	27	Long-term administration of clopidogrel to patients with atherosclerotic vascular disease is more effective than aspirin in reducing the combined risk of ischemic stroke, myocardial infarction, or vascular death
CHARISMA (PAD subgroup)	RCT, double-blind, 2 × 2 factorial, placebo-controlled, multicenter	28 months	Patients with PAD identified in CHARISMA study. Current intermittent claudication + an ABI ≤ 0.85, or a history of intermittent claudication + previous related intervention (amputation, surgical or catheter-based peripheral revascularization)	3096	70	30	Among patients with PAD, the primary end point occurred in 7.6% in the clopidogrel plus aspirin group and 8.9% in the placebo plus aspirin group (*p* = 0.18). The rate of MI and hospitalization for ischemic events were lower in the DAPT arm than aspirin alone
EUCLID	Double-blind, event-driven RCT	30 months	50 years of age with symptomatic peripheral artery disease. One of two inclusion criteria: previous revascularization of the lower limbs for symptomatic disease more than 30 days before randomization or hemodynamic evidence of peripheral artery disease	13,885	72	28	The primary efficacy end point occurred in 10.8% receiving ticagrelor and in 10.6% receiving clopidogrel failing to show ticagrelor to be superior to clopidogrel for the reduction of cardiovascular events (*p* = 0.65)
COMPASS	Double-blind double-dummy RCT using a 3-by-2 partial factorial design	23 months	Adults who meet criteria for CAD, PAD or both	27,395	78	22	Combination therapy with rivaroxaban (2.5 mg twice daily) plus aspiring among patients with stable atherosclerotic vascular disease had statistically significant better cardiovascular outcomes and more major bleeding events than those assigned to aspirin alone
4S	Double-blind RCT	5.4 years	Adults 35–70 years with history of angina pectoris or MI	4444	81.3	18.7	Simvastatin produced significant reduction of cardiovascular mortality in patients with CAD. Probability of a woman > 60 years escaping a major coronary event was 77.7% in placebo and 85.1% in simvastatin arm (*p* = 0.01). RR of death or coronary event in women < 60 were 0.63 and 0.61, respectively
WOSCOPS	Double-blind RCT	4.9 years	Fasting LDL > 155; no history of MI, arrhythmia or other serious illness, men with stable angina who had not been hospitalized within the previous 12 months	6595	100	0	Pravastatin lowered plasma cholesterol levels by 20% and low-density lipoprotein cholesterol levels by 26%. A 22% reduction in the risk of death from any cause in the pravastatin group was observed
HOPE	Double-blind, 2 × 2 factorial, RCT	3.5 years	Adults > 55 years old with history of CAD, PAD, CVA, or DM + another CV risk factor	9297	73	27	Treatment with ramipril-reduced rates of death from cardiovascular causes, MI, stroke, death from any cause, revascularization procedures, heart failure and complications related to DM
CAMELOT	Multicenter, double-blind, placebo-controlled RCT	24 months	Adults 30–79 years old requiring coronary angiography for evaluation for chest pain or percutaneous coronary intervention + DBP < 100 with or without treatment	1991	73.7	26.3	Administration of amlodipine to patients with CAD and normal blood pressure resulted in reduced adverse cardiovascular events particularly in women (RRR 42.8%) + IVUS showed evidence of slowing of atherosclerosis progression
FOURIER (PAD subgroup)	Double-blind RCT	2.2 years	Clinically evident atherosclerotic cardiovascular disease including prior MI, prior ischemic stroke, or symptomatic PAD (intermittent lower extremity claudication and an ankle-brachial index < 0.85, a history of a peripheral artery revascularization procedure, or a history of amputation attributable to atherosclerotic disease)	3642	71.8	28.2	ARR for CV death, MI, or stroke 3.5% in patients with PAD, and 1.4% in patients without PAD
STOP-IC	Prospective RCT, open-label, multicenter	12 months	Patients with symptomatic PAD attributable to de novo femoropopliteal lesions	191	68.5	31.5	The angiographic restenosis rate at 12 months was 20% in the cilostazol group in comparison with 49% in the noncilostazol group (*p* = 0.001; odds ratio, 0.26; 95% confidence interval, 0.13–0.53)
Exercise therapy							
CLEVER	Multicenter RCT across 29 centers in US and Canada	18 months	Adults > 40 years of age with moderate to severe claudication due to aortoiliac PAD. *Moderate to severe* claudication was defined as the ability to walk at least 2 min on a treadmill at 2 miles per hour at no grade, but <11 min on a graded treadmill test using the Gardner-Skinner protocol	111	62	38	Supervised exercise provides a superior improvement in treadmill walking performance compared to both primary aortoiliac revascularization and optimal medical care (home walking and cilostazol) over 6 months (*p* < 0.001 for the comparison of SE versus OMC, *p* = 0.02 for ST versus OMC, and *p* = 0.04 for SE versus ST). This benefit was also associated with an improvement in self-reported walking distance, an increase in high-density lipoprotein, and a decrease of fibrinogen. Secondary measures of treatment efficacy favored primary stenting, with greater improvements in self-reported physical function
ERASE	Parallel-design RCT conducted in the Netherlands at 10 sites	12 months	PAD and stable claudication (≥ 3 months) with a resting ABI of < 0.90 or if their ABI decreased by more than 0.15 after treadmill testing regardless of their ABI at rest. All participants also had 1 or more vascular stenoses at the aortoiliac level, the femoropopliteal level, or both. Maximum walking distance had to be between 100 m and 500 m	666	62	38	Combination therapy of endovascular revascularization followed by supervised exercise resulted in significantly greater improvement in walking distances and health-related quality of life scores compared with supervised exercise only
Interventional							
IN.PACT SFA	prospective, multicenter, single-blinded, RCT	12 months	Patients with intermittent claudication or ischemic rest pain due to superficial femoral and/or popliteal PAD	331	66	34	Drug-coated balloon was superior to PTA and had a favorable safety profile for the treatment of patients with symptomatic femoropopliteal peripheral artery disease
LEVANT-2	Single-blind, RCT	12 months	Rutherford stage 2–4 with ≥ 70% angiographically significant atherosclerotic lesion in the superficial femoral or popliteal artery, or both. The total treated lesion length had to be 15 cm or less, and the reference diameter of the target vessel had to be 4–6 mm	476	63	37	PTA with a paclitaxel-coated balloon resulted in a rate of primary patency at 12 months that was higher than the rate with angioplasty with a standard balloon
THUNDER	RCT, multicenter	5 years	Symptomatic peripheral artery disease with one or more obstructive lesions, either new lesions or restenoses, at least 70% of vessel diameter and at least 2 cm in length, in the superficial femoral artery, the popliteal artery, or both	154	65.5	34.5	Use of paclitaxel-coated angioplasty balloons (PCB) during percutaneous treatment of femoropopliteal disease is associated with significant reductions in late lumen loss and target lesion revascularization. Reduced rate of revascularization following PCB treatment was maintained over a 5 year period, although noted to be higher in women
ABSOLUTE	Single-institution RCT	12 months	Symptomatic PAD with Rutherford stage 3–5; > 50% or occlusion of the ipsilateral superficial femoral artery, a target lesion length of more than 30 mm, and at least one patent (< 50% stenosis) tibioperoneal runoff vessel	104	53	47	At 6–12 months, treatment of superficial femoral artery disease by primary implantation of a self-expanding nitinol stent yielded results that were superior to those with the currently recommended approach of balloon angioplasty with optional secondary stenting
ASTRON	Multicenter RCT	12 months	Symptomatic PAD Rutherford class 3–5; > 50% stenosis or occlusion of the SFA with a target lesion length between 30 mm and 200 mm, and at least one patent (< 50% stenosis) tibioperoneal runoff vessel	73	68	32	Primary stenting with a self-expanding nitinol stent for treatment of intermediate length SFA disease resulted morphologically and clinically superior midterm results compared with balloon angioplasty with optional secondary stenting
FAST	Multicenter RCT in 11 European centers	12 months	De novo SFA lesion located at least 1 cm from the SFA origin with a length between 1 and 10 cm. Target lesion diameter stenosis had to be at least 70% by visual estimate. The popliteal artery as well as 1 of the infrapopliteal (below-the-knee) vessels had to be continuously patent for sustained distal runoff. Clinically, patients to have at least Rutherford category 2	244	69	31	No statistically significant difference between treatment groups was observed at 12 months in the improvement by at least 1 Rutherford category of peripheral arterial disease
PACIFIER	Investigator-initiated multicenter RCT conducted in three German institutions	12 months	Claudication or critical limb ischemia (Rutherford 2–5); disease of SFA or popliteal artery; lesion length 3–30 cm; an occlusion or a grade of stenosis ≥ 70%, and absence of contraindications to dual antiplatelet therapy	85	59	41	DEB was associated with significant reductions in late lumen loss and restenosis at 6 months, and re-interventions after femoropopliteal percutaneous transluminal angioplasty up to 1 year of follow-up
VIASTAR	Prospective, single-blind, multicenter, RCT	24 months	Symptomatic PAD in the Rutherford stage 2–5, de novo arteriosclerotic stenosis or occlusion of the SFA and proximal popliteal artery 10–35 cm in length (TASC II classes B-D), patent or successfully treated iliac artery inflow, and outflow of at least 1 tibial artery	141	71	29	In lesions ≥ 20 cm, (TASC class D), the 12-month patency rate was significantly longer in VIA patients. Freedom from target lesion revascularization was 84.6% for Viabahn versus 77.0% for BMS. The ankle-brachial index in the Viabahn group significantly increased compared with the BMS at 12 months
DEBATE-BTK	Single-center, parallel-group, open blinded end point RCT	12 months	presence of diabetes mellitus, CLI (Rutherford class 4 or greater), stenosis or occlusion ≥ 40 mm of at least 1 tibial vessel with distal runoff to the foot, and agreement to 12-month angiographic evaluation	132	80	20	Drug-eluting balloons compared with PTA strikingly reduce 1-year restenosis, target lesion revascularization, and target vessel occlusion in the treatment of below-the-knee lesions in diabetic patients with critical limb ischemia
ZILVER	Prospective, multinational RCT with a complementary single-arm study	24 months	Rutherford category ≥ 2, ≥ 50% diameter stenosis, reference vessel diameter 4–9 mm, lesion length up to 14 cm, and at least 1 patent runoff vessel with < 50% stenosis throughout its course	474	65	35	Primary DES group demonstrated significantly superior 2-year event-free survival and primary patency
FEMPAC	Multicenter RCT	6 months	Occlusion/stenosis ≥ 70% diameter of the SFA and/or popliteal artery with clinical Rutherford stages 1–5; successful guidewire passage of the lesion during angiography	87	60	40	The number of target lesion revascularizations was lower in the paclitaxel-coated balloon group than in control subjects (*p* = 0.002). Improvement in Rutherford class was greater in the coated balloon group (*p* = 0.045), whereas the improvement in ankle-brachial index did not achieve statistical significance
BASIL	Multicenter RCT, prospective, across 27 UK hospitals	5.5 years	Severe limb ischemia, for > 2 weeks, and who on diagnostic imaging had a pattern of disease which, in joint investigator opinions, could equally well be treated by either infra-inguinal bypass surgery or balloon angioplasty	452	59.5	40.5	In patients presenting with severe limb ischemia due to infra-inguinal disease and who are suitable for surgery and angioplasty, a bypass surgery-first and a balloon angioplasty-first strategy are associated with broadly similar outcomes in terms of amputation-free survival, and in the short-term, surgery is more expensive than angioplasty
ACHILLES	Prospective multicenter RCT in nine European countries	1 year	Adults with infrapopliteal PAD. Reasons for exclusion were significant stenoses (> 50%) distal to the target lesion that might require revascularization or impede runoff; angiographically evident thrombus or history of thrombolysis within 72 h; untreated lesions (> 75% stenosis), Cr > 2.5 mg/dl	200	71.5	28.5	lower angiographic restenosis rates (22.4 vs 41.9%, *p* = 0.019), greater vessel patency (75.0% vs 57.1%, *p* = 0.025), and similar death, repeat revascularization, index-limb amputation rates, and proportions of patients with improved Rutherford class for sirolimus-eluting stents vs PTA
DESTINY	RCT, multicenter European	12 months	Symptomatic PAD due to a maximum of two focal de novo atherosclerotic target lesions in one or more infrapopliteal vessels	140	63.5	36.5	Treatment of the infrapopliteal occlusive lesions of CLI with everolimus stents demonstrated an 85% patency vs 54% with BMS at 12 months, decrease in restenosis, as well as statistically significant independence from revascularization
YUKON-BTX	Double-blind RCT	12 months	Rutherford class 3–5, presence of a single primary target lesion in a native infrapopliteal artery that was 2.5–3.5 mm in diameter and that did not exceed 45 mm in length	161	66.5	33.5	BMS placement was associated with a hazard ratio for restenosis of 3.2 (95% CI 1.5 to 6.7; *p* = 0.003) compared with sirolimus-eluting stents (SES) after 1 year. No significant differences between the study groups concerning mortality and amputation rates were observed, but mean ABI and Rutherford scores showed significant improvements in sirolimus group
IN.PACT DEEP CLI	Prospective multicenter RCT	12 months	Rutherford class 4–6 symptomatic CLI patients; reference vessel diameters between 2 and 4 mm; single or multiple lesions with ≥ 70% stenosis of different lengths in one or more main afferent crural vessels including tibioperoneal trunk	358	74.3	25.7	IN.PACT Amphirion drug-eluting balloons demonstrated comparable and non-inferior efficacy to PTA in CLI patients. The overall complication rate, a composite of core laboratory-adjudicated incidence of vasospasm, abrupt closure, vessel recoil, thrombus, and perforation, was higher in the IA-DEB arm versus the PTA arm (9.7 vs 3.4%; *p* = 0.035). Major amputation-free survival had a trend favoring DEB
IDEAS	Prospective RCT	6 months	Rutherford classes 3–6 and angiographically documented infrapopliteal disease with a minimum lesion length of 70 mm	50	76	34	DES are related with significantly lower residual immediate post-procedure stenosis and have shown significantly reduced vessel restenosis at 6 months

## Epidemiology and Risk Factors

Women with PAD present on average 10–20 years later than men [20]. Around 20–30% of women aged 70 years or older are affected by PAD [21, 22]. This is hypothesized to be secondary to the loss of the vascular protective effects of estrogen which promotes vasodilation and has anti-oxidative effects. In a study of > 370,000 surgical inpatients with PAD, Vouyouka et al. found that women were more likely to be older, obese, and black [23•]. Overall risk factors for PAD remain similar among men and women, including smoking, age, diabetes mellitus, hypertension, and dyslipidemia [3]. Diabetes and hyperlipidemia have been shown to increase the risk of intermittent claudication by fourfold in women [24, 25]. Importantly, ethnic differences have been shown to affect the prevalence of PAD as well, with the highest prevalence of PAD among non-Hispanic black women over the age of 70 (25%) [26•]. Other studies have shown association between obesity [27], levels of C-reactive protein (CRP) [28, 29], osteopenia/osteoporosis [30, 31], hypothyroidism, and PAD. In the Multiethnic Study of Atherosclerosis (MESA) [29], women had higher levels of CRP than men, after adjustment for comorbidities, hormonal status, and age. Conflicting data has emerged for the association between hormone replacement therapy and PAD in women. In the Women Health Initiative (WHI) and Heart and Estrogen/Progestin replacement (HERS) studies, no benefit was observed from HRT use for PAD or CAD. Conversely, the Rotterdam study showed a 52% decreased risk of PAD in women who used HRT for > 1 year [32–34]. Interestingly, vascular complications associated with pregnancy have also been associated with an increased risk of PAD. The Cardiovascular Health After Maternal Placental Syndrome (CHAMPS) study showed a threefold increased risk of PAD and twofold increased risk of coronary artery and cerebrovascular disease in patients with maternal placental syndromes, including pre-eclampsia, gestational hypertension, placental abruption, and placental infarction [35]. The mechanisms for this association are unclear, although one likely hypothesis is underlying endothelial dysfunction.

The treatment of risk factors varies by gender. In the REACH registry [4], consisting of > 68,000 outpatients, risk factor control was less frequently observed in patients with diagnosis of PAD. Optimal risk factor control was twice as likely for men than women despite a higher incidence of diabetes, hypertension, and elevated total cholesterol in women.

## Symptoms

Both men and women present with typical, atypical, or asymptomatic PAD. Studies have shown that the majority of PAD patients do not have typical claudication [11, 36]. Asymptomatic disease is defined as absence of exertional leg symptoms in the presence of an ankle-brachial index (ABI) < 0.90, while atypical symptoms are defined by leg symptoms present at rest and exercise [37, 38]. In the Women Health and Aging study (WHAS), of the 933 women enrolled, 35% (*n* = 328) had an ABI of 0.90; of these, 328 patients (63%) had no exertional leg symptoms [39]. Importantly, asymptomatic PAD has been shown to be more common in women than in men (13 vs 9%; *p* < 0.03) [40]. When symptomatic, women seek medical attention with more complex (multilevel) and severe disease including critical limb ischemia (CLI) [16•, 40, 41]. In patients with CLI, women had a twofold higher incidence of femoropopliteal disease compared to men [42]. This finding was reproduced in another patient cohort undergoing angioplasty that showed pronounced femoropopliteal disease in women while men had more below-the-knee disease [43]. Additionally, women have greater lower extremity functional impairment [8], with shorter treadmill distance to intermittent claudication [44], shorter maximal treadmill walking distance [8, 44], and poorer quality of life scores compared to men [45]. Other studies have demonstrated a higher prevalence of asymptomatic disease in women, which may lead to a late presentation, thus contributing to severe disease or CLI [41].

## Treatment

The principle components of PAD treatment consist of supervised exercise therapy, pharmacological treatment, and lower extremity revascularization. Patients with PAD are less likely to receive guideline-directed medical therapy (GDMT) than are patients with other forms of cardiovascular disease, including CAD [46–48]. For example, in one study on secondary prevention of PAD, statin use was reported in only 31%, angiotensin-converting enzyme inhibitor use in 25%, and aspirin use in 36% [48]. Data also exists on suboptimal use of systemic vascular treatment or lack of adherence to standard therapy. In the NHANES study, only 24–34% adherence to preventive therapy was reported [48]. CHAMPS study cited similar suboptimal use of GDMT but was particularly notable for lower rates in women and older patients [31]. In terms of intensity of treatment with standard pharmacologic agents, men were more likely to receive all agents (antiplatelets, statins, and angiotensin enzyme inhibitors) than women (22.4 vs 18.2%) [31]. This finding was reproduced in another study from Quebec which showed that men were more likely to receive statins, antiplatelet agents, and angiotensin-converting enzyme inhibitors than women (22.4 vs 18.2%, *p* < 0.001) [31].

Patients with PAD experience a profound limitation in exercise performance. There is evidence of a well-established benefit following a typical 12-week exercise training program [49, 50]. Lower extremity exercise training has been shown to increase time to claudication, increase distance before claudication, and increase overall walking distance [51]. Unfortunately, women with PAD have been shown to be less responsive to exercise rehabilitation programs [52], particularly diabetic women. This may partly be due to a greater impairment in calf muscle oxygen saturation during and following exercise [53]. Gardner et al. reported that improvements in absolute walking distance were significantly less for women than men after 1 year of standard exercise therapy. Women also reported less subjective improvement on walking impairment questionnaire domains [54]. These differences have been attributed to lower hemoglobin saturation during ambulation [53], poorer leg strength [55], higher inflammation, higher level of oxidative stress, and insulin resistance [53].

Endovascular revascularization and open bypass surgery are two strategies for disabling claudication after failure of medical therapy or for those with CLI. Although the choice of procedure depends on many lesion characteristics including lesion site [56, 57], the 2016 AHA/ACC Guidelines recommend an endovascular approach first for both lifestyle limiting claudication and CLI. While data has been equivocal, sex differences have also been reported in lower extremity endovascular versus bypass treatment. Using 69 million discharge records from the Nationwide Inpatient Sample from 1998 to 2006, Roe et al. reported discrepancies in the proportion of endovascular procedures being performed in women compared to men. Women were less likely to undergo amputation or open vascular surgery than men. Women, however, were more likely to undergo an endovascular procedure during hospitalization [58, 59]. Several possible reasons have been cited for lower bypass rates, including the observation that women with PAD are generally older with more advanced disease, comorbidities, and may have smaller vessel size precluding bypass.

## Carotid Artery Stenosis and Management

Women have a greater risk of disabling stroke (58 vs 48%) and stroke-related mortality (20 vs 14%) [60]. Stroke-related mortality has not changed over the past 50 years in women and is attributed to older age at onset of stroke among women [60]. Multiple trials have demonstrated a reduction in the risk of stroke in select patients with symptomatic internal carotid artery disease and to a lesser extent, in those with asymptomatic carotid artery disease [61–63]. However, it is noteworthy that women comprised only 28–34% of enrolled patients in these trials. In an analysis of the North American Symptomatic Carotid Endarterectomy Trial (NASCET) and ACAS trial, 30-day risk for death was higher in women than in men (2.3 vs 0.8%, *p* = 0.002), owing to higher risk of fatal stroke [64]. Both men and women benefited from carotid endarterectomy (CEA) for stroke prevention. However, in another study, the risk of stroke or death within 30 days after CEA in symptomatic patients was greater in women (8.7%) vs men (6.8%) [65], a finding which was reproduced in a systematic review of 36 studies [66]. However, other studies have shown no significant difference in complications and mortality following CEA [67, 68]. Regarding carotid artery stenting, women have worse outcomes, including higher rates of in-hospital mortality and stroke [69]. Risk of stroke or mortality was 1.7-fold higher in symptomatic women and 3.4-fold higher in asymptomatic women with carotid artery stenosis (CAS) compared to CEA. Asymptomatic women experienced worse outcomes compared to men, with higher stroke rates after CEA and higher myocardial infarction rates after both CEA and CAS [70].

## Quality of Life

Quality of life scores have become an important tool to assess treatment effectiveness in the general population. Multiple studies have shown worse health status and health-related quality of life in women when compared with men suffering from PAD [10, 12, 13]. In addition, functional status has been determined to be significantly lower for women [45]. This was associated with greater mood disturbances [12]. Female gender has been adversely associated with durability of the revascularization or the quality of life following revascularization for claudication or CLI [71]. In a longitudinal study of a large PAD population, women with PAD were found to have compromised health status both at diagnosis and 12 months after follow-up. The mechanism for poor health status in these women was thought to be associated with lower education and lack of social support (women were less likely to have a partner) [72].

## Outcomes/Prognosis

Outcome trials of endovascular or surgical revascularization in men and women have reported conflicting results. Several studies have reported an unfavorable impact of sex on outcomes after peripheral revascularization procedures Women tend to have higher perioperative mortality whether undergoing surgical or endovascular procedures [73, 74]. Furthermore, they have inferior patency rates after surgical revascularization [75–77], higher risk of stent thrombosis with endovascular revascularization [77], wound complications [78], and bleeding events [23•]. On the other hand, multiple other studies, including some systematic reviews, have found no sex difference in patency rates and amputation-free survival [79–81].

PAD is associated with increased risk of CVD mortality and morbidity. A low ABI (≤ 0.9) is associated with a threefold increased risk of all-cause mortality and cardiovascular mortality in both men and women [82]. Women with PAD have a two- to fourfold increased risk of cardiovascular mortality and morbidity compared to women without PAD [15]. Compared to men, women are more likely to be admitted for acute myocardial infarction [83], more likely to be admitted emergently with longer hospital stays and more likely to require rehabilitation or nursing home care [16•, 59, 84]. Similarly, women with CLI have higher in-hospital mortality after both endovascular treatments and open surgery [85].

## Conclusions

PAD remains a major healthcare problem. It remains underdiagnosed and understudied in women. The major challenge in PAD treatment in women is their late presentation and the higher prevalence of asymptomatic disease which may lead to more advanced disease at presentation and a higher risk of adverse events and mortality. Concerted research efforts should be carried out to further determine the effects of sex on different aspects of PAD including risk factors, clinical burden, treatment, and outcomes. In addition, campaigns to raise awareness among clinicians and the general public should be undertaken. Efforts along the lines of the “National Wear Red Day” campaign by the AHA should be pursued aggressively to increase awareness.
